# Corrigendum

**DOI:** 10.1111/crj.13562

**Published:** 2023-01-09

Meng, X , Lin Q , Yin H , Li Z . Hyperpolarized ^3^helium MRI measured apparent diffusion coefficient and its correlations with pulmonary functions tests in patients with chronic obstructive pulmonary disease: a meta‐analysis. Clin Respir J 2021;15:1185–1193.3428850510.1111/crj.13425

In the above article, the below funding statement should be added.

This work was supported by the Scientific Research Project of Heilongjiang Provincial Health Commission (2020‐245).

The authors apologize for these errors.

